# STR-Uggles: Overcoming Humic Acid Inhibition Using Combined STR & qPCR Kit Chemistries

**DOI:** 10.3390/genes16111326

**Published:** 2025-11-03

**Authors:** Caitlin McDonald, Duncan Taylor, Adrian Linacre

**Affiliations:** 1 College of Science and Engineering, Flinders University, Adelaide, SA 5001, Australiaduncan.taylor@sa.gov.au (D.T.); 2 Forensic Science SA, Adelaide, SA 5001, Australia

**Keywords:** inhibition, humic acid, STR DNA profile

## Abstract

Background/Objectives: DNA profiling can fail, or produce poor results, when naturally occurring materials are present during the amplification step. This study demonstrates that simple modifications to the reaction setup can overcome this obstacle. PCR inhibition is caused by a range of compounds including haem, humic acid and dyes. Various strategies to overcome this inhibitory effect have been explored, such as improving extraction methods to remove these compounds, diluting samples to reduce inhibitor concentration, or using inhibitor-tolerant DNA polymerases. In this study, we evaluate whether modified setups can help mitigate the effects of humic acid, a common inhibitor that induces various inhibition mechanisms. Methods: We combined the GlobalFiler STR kit with Investigator Quantiplex Pro into a single reaction. Supplementing the amplification GlobalFiler with additional reagents creates altered amplification environments that use additional DNA polymerase and reaction buffer. Results: The modified setups outperformed the standard GlobalFiler protocol, even at the highest concentration of humic acid tested. The STR reactions supplemented with qPCR reagents produced higher-quality profiles with improved allele amplification and an even peak balance, indicating that a dual-DNA polymerase system offers a more robust and inhibitor-tolerant environment for STR amplification. In addition to demonstrating the value of this combined approach, these data provide a comprehensive dataset characterising the impact of increasing humic acid concentrations on profile quality from an ideal DNA input. Conclusions: For PCR inhibitors with similar mechanisms this approach offers broader applicability in forensic casework and a promising step toward more reliable and robust profiling of inhibited samples.

## 1. Introduction

Despite the many advancements in DNA profiling processes since their introduction in the 1980s [1], inhibition remains a significant challenge for forensic scientists. However, this study demonstrates that simple alterations to the reaction setup can maintain profile quality under inhibitory pressure. This challenge is perhaps most prominent when analysing samples affected by inhibition that have the additional complexity that they are in trace quantities, degraded, or exposed to non-ideal environmental conditions. The presence of inhibitory compounds during the polymerase chain reaction (PCR) can impair DNA polymerase activity, which often means sub-optimal or partial profiles are produced or amplification fails entirely. In a short tandem repeat (STR) profile, this inhibition manifests as allelic imbalance, allelic dropout, increased artefactual peaks and stochastic effects, and greater variability between replicates of the same sample [2,3]. Thus, inhibition reduces the overall quality and interpretability of DNA profiles, which in turn limits their value in forensic investigations.

A wide range of compounds are known to inhibit DNA amplification during PCR, many of which are also commonly encountered in samples submitted for forensic DNA analysis. These include humic acid (present in soil), haem (a component of blood), indigo dyes (from denim and other clothing), tannins (found in wood, bark and certain fruits), collagen (present in food products and human tissues), bile salts (from faecal matter) and silicon-based compounds (commonly found on condoms and personal lubricants) [4,5,6,7,8]. Inhibition of DNA polymerase, whether through direct interaction with the enzyme or through interference with cofactors, such as magnesium ions (Mg^2+^) [9], compromises the amplification of STR targets and thus, the quality of resulting DNA profiles.

These inhibitors are frequently present in combinations within samples. For example, a bloodstained item of clothing recovered from a violent assault may contain haem, collagen, and textile dyes, each of which contribute to a large, cumulative inhibitory effect. In such cases, even samples with high-quality DNA in large amounts may fail to produce an informative DNA profile. The challenge of inhibited samples is further compounded by the variability in both DNA quantity and inhibitor concentration across different sample types. Whether dealing with trace DNA and low levels of inhibitors, or high-quality DNA and substantial inhibition, the impact of these compounds on DNA amplification and profiling success can be substantial [10,11,12,13,14,15,16]. Furthermore, the complexity of inhibited samples presents a significant burden for operational forensic laboratories, where considerable time and resources are invested in processing inhibited samples with little to no return of useable genetic data. As such, any strategies that improve DNA profile quality in the presence of inhibitors have the potential to significantly reduce wasted resources and enhance casework outcomes.

The inhibitory effects of various compounds on the PCR process are well-documented, with inhibitory substances typically classified into three main categories: DNA polymerase inhibitors, nucleic acid inhibitors, and fluorescence quenchers [4,17], although the latter is not technically an inhibition of the PCR process. These classifications are largely based on the primary mechanism through which inhibition occurs for each substance, however, some compounds exhibit inhibitory effects across multiple categories. Humic acid, for example, is known to interfere with DNA polymerase activity, bind to nucleic acids, and quench fluorescence signals which makes it a particularly challenging inhibitor in forensic DNA analysis [4,6,7,17,18,19]. Given its ability to disrupt multiple aspects of the PCR process, humic acid provides an ideal compound for investigating methods to overcome PCR inhibition. By affecting all major components of STR amplification for DNA profiling, the use of humic acid allows for a more comprehensive assessment of methods designed to improve DNA profile quality under inhibited conditions. Additionally, the extensive literature on the inhibitory mechanisms of humic acid [4,6,17,20] and its frequent occurrence in samples submitted to operational forensic laboratories further support its selection as a representative inhibitor for this study.

In recent years there been research into potential ways to overcome inhibition, including optimisation of DNA purification and extraction techniques [17,21,22], dilution of samples to reduce inhibitor concentration [23,24], using an alternative polymerase [25], blending polymerases [26] and the development of inhibitor-tolerant mutant DNA polymerases [27]. However, the success of DNA profiling such samples remains low. We explore an alternative approach that utilises one or two readily available amplification chemistries combined to overcome inhibition.

In previous work we have explored the use of a combined endpoint PCR and qPCR setup that allows real-time monitoring of amplification alongside amplification of STR targets for DNA profiling [28,29,30]. This approach has been found to be successful using ideal and trace DNA samples (to the level that some combined kit chemistry reactions outperformed standard endpoint kit chemistry alone); however, such an approach also may help to overcome inhibition. The combined reaction approach has two benefits that may help address current inhibition issues: the first being that it will allow amplification efficiency to be monitored, but the second and perhaps the most important, is that it uses a combination of DNA polymerases and reaction buffers. The use of two polymerases and reaction buffers creates a unique environment for PCR to occur—one where in theory, amplification can be enhanced, which may allow amplification to occur more effectively thus allowing more informative DNA profiles to be obtained from inhibited samples—where current operational workflows fall short. It is the trailing of combinations of differing buffer chemistries and polymerases that distinguished our study from those previously mentioned.

This study aimed to evaluate if integrating endpoint STR and real-time qPCR chemistries can mitigate the effects of humic acid inhibition and enhance the quality of DNA profiles. This will be achieved by combining the components of both GlobalFiler and Investigator Quantiplex Pro in the same reaction.

## 2. Methods

### 2.1. Ethics Approval

Ethics approval for this project was obtained from the Low-Risk Human Research panel of the Social and Behavioural Research Ethics Committee at Flinders University, Australia (reference number 4915). All DNA samples provided by the volunteer were obtained with informed consent.

### 2.2. Collection of Control DNA

Cellular material was collected from a consenting donor via 10 buccal swabs (inner cheek) and saliva samples. The buccal swabs were collected using a 4N6FLOQSwab^TM^ (ThermoFisher Scientific, Melbourne, VIC, Australia). DNA was extracted from all samples using the QIAamp DNA Investigator System^TM^ (QIAGEN, Clayton, VIC, Australia) following the manufacturer’s protocol with a final elution volume of 40 μL. The 10 extracts were then combined to produce a 400 μL homogenised sample which was then quantified using the Investigator Quantiplex Pro RGQ Kit^TM^ (QIAGEN) and Rotor-Gene Q system (QIAGEN) following the validated manufacturer’s protocol. The data were analysed using the QIAGEN Quantification Assay Data Handling Tool. This DNA extract was diluted in Amplification Grade Water (Promega, Sydney, NSW, Australia) and used as control DNA for all experiments detailed in this study.

### 2.3. Inhibitor Stock Preparation

Stock solutions of humic acid (10 g, Merck, Bayswater, VIC, Australia) were prepared in Amplification Grade Water (Promega) at concentrations of 50, 100, 200 and 300 ng/μL for use in this study. As humic acid is only partially soluble in water, all solutions were vortexed thoroughly to achieve homogeneity after preparation and were re-vortexed immediately prior to pipetting to minimise precipitation.

### 2.4. DNA Amplification

#### 2.4.1. Standard Endpoint PCR Setups

Standard endpoint PCR setups were prepared as a baseline for comparison with inhibited samples. Each standard reaction contained 10 μL of GlobalFiler^TM^ (ThermoFisher Scientific) Master Mix (comprised of 7.5 μL of master mix and 2.5 μL of primer mix), 14 μL of control DNA at 0.0357 ng/μL (500 pg total DNA), and 1 μL of Amplification Grade Water (Promega), for a total reaction volume of 25 μL. Inhibited samples were prepared similarly except that the 1 μL of Amplification Grade Water was replaced with 1 μL of humic acid stock at concentrations of 50, 100, 200 and 300 ng/μL. Five replicates of each setup were amplified, totalling 25 reactions. All amplifications followed the validated GlobalFiler^TM^ manufacturer’s protocol for 30 cycles and were performed on the Open-Source qPCR Thermal Cycler (Chai Biosystems, Santa Clara, CA, USA). The amplified DNA was then separated on a 3500 Genetic Analyser^TM^ (ThermoFisher Scientific) using 8.5 μL Hi-Di Formamide, 0.5 μL 600 LIZ^®^ Size Standard (ThermoFisher Scientific) and 1 μL of the amplified PCR product.

#### 2.4.2. Combined qPCR and Endpoint PCR Setups

Combined qPCR and STR setups were also prepared for all inhibited samples to allow comparison between profile quality of the standard GlobalFiler and combined reaction profiles. These combined qPCR and STR setups were composed of a full-volume GlobalFiler^TM^ reaction (7.5 μL of master mix and 2.5 μL of primer mix) and a half-volume Investigator Quantiplex Pro reaction (Qiagen) (4.5 μL of reaction mix and 4.5 μL of primer mix) as outlined in previous work [20,21], in addition to 5 μL of control DNA at 0.1 ng/μL (500 pg total DNA), and 1 μL of Amplification Grade Water, for a total reaction volume of 25 μL. As with the standard endpoint setups, for the combined setups the inhibited samples used 1 μL of humic acid stock at concentrations of 50, 100, 200 and 300 ng/μL in place of the 1 μL of Amplification Grade Water. Five replicates of each combined setup were amplified, totalling 25 reactions. All amplifications followed the validated GlobalFiler^TM^ manufacturer’s protocol for 30 cycles and were performed on the Open-Source qPCR Thermal Cycler.

#### 2.4.3. Standard Endpoint PCR with Additional Taq Polymerase and Buffer

To investigate whether the addition of more polymerase and buffer from the same STR kit improved DNA profile quality under inhibition, standard GlobalFiler^TM^ setups were supplemented with additional master mix. This allowed direct comparison between the profile quality of the standard GlobalFiler^TM^ reactions and those with increased reagent volumes.

These samples were composed of 14.25 μL of GlobalFiler^TM^ master mix, 4.75 μL of GlobalFiler^TM^ primer mix, in addition to 5 μL of control DNA at 0.1 ng/μL (500 pg total DNA), and 1 μL of Amplification Grade Water, for a total reaction volume of 25 μL. As with the previous setups in this study, the inhibited samples used 1 μL of humic acid stock at concentrations of 50, 100, 200 and 300 ng/μL in place of the 1 μL of Amplification Grade Water. Five replicates of each combined setup were amplified, totalling 25 reactions. All amplifications followed the validated GlobalFiler^TM^ manufacturer’s protocol for 30 cycles and were performed on the Open-Source qPCR Thermal Cycler.

#### 2.4.4. Standard Endpoint PCR with qPCR Taq Polymerase and Buffer

To investigate if the use of additional polymerase and buffer from a different STR kit was more effective at overcoming inhibition than the addition of more from the same kit (as performed in Section 2.4.3), standard GlobalFiler^TM^ setups were also supplemented with Investigator Quantiplex Pro reaction mix (i.e., not including the primer mix). This allowed comparison of DNA profile quality across the four different reaction setups.

These reactions were composed of a standard full-volume GlobalFiler^TM^ reaction (7.5 μL of master mix and 2.5 μL of primer mix), supplemented with 4.5 μL of Investigator Quantiplex Pro reaction mix, 9.5 μL of control DNA at 0.053 ng/μL (500 pg total DNA), and 1 μL of Amplification Grade Water, giving a total reaction volume of 25 μL. As with other setups in this study, inhibited samples used 1 μL of humic acid stock at concentrations of 50, 100, 200 and 300 ng/μL in place of the 1 μL of Amplification Grade Water. Five replicates of each combined setup were amplified, totalling 25 reactions. All amplifications followed the validated GlobalFiler^TM^ manufacturer’s protocol for 30 cycles and were performed on the Open-Source qPCR Thermal Cycler.

### 2.5. STR Profiling

All amplified DNA was separated on a 3500 Genetic Analyser^TM^ (ThermoFisher Scientific) using 8.5 μL Hi-Di Formamide, 0.5 μL LIZ^®^ 600 Size Standard (ThermoFisher Scientific) and 1 μL of amplified PCR product. The settings used for the 3500 Genetic Analyzer were 1.2 kV/15 s injection and 13 kV/1550 s runtime with dye set J6 (ThermoFisher Scientific).

### 2.6. Data Analysis

STR data were analysed using GeneMapper^®^ ID-X (version 1.4) with the heterozygous and homozygous allele calling thresholds set to 50 relative fluorescence units (RFU) and 100 RFU, respectively. In previous work [28,30] we defined a metric to score DNA profile quality. In this study we present a refined version of this metric which incorporates a transformation of the mean peak height to better reflect the acceptable range of values observed in DNA profiles.

Previously the “ideal” average peak height (i.e., the total summed allelic RFU divided by the number of diploid loci only) was set to 5000 RFU [28] and many profiles produced under modified PCR conditions (different kit chemistries) were mildly penalised as they consistently exhibited average peak heights in the range of 7000–8000 RFU. While these values are beyond the defined ideal range, they do not in practice indicate any loss in profile quality and are not associated with signal saturation, increased pull-up, or other capillary electrophoresis (CE) related artefacts. Using the original metric these profiles incurred penalties that masked their otherwise acceptable or even improved quality.

We transform $\bar{x}$ to ${\bar{x}}_{adj}$ so that the peak height penalty is constant for all profiles with peak heights in the range of 5000 to 10,000 RFU. This ensures that profiles with higher but still desirable peak heights are not unfairly scored lower. The choice of 10,000 RFU as an upper bound was partly based on author experience in reading DNA profiles but also based on the fact that a homozygous peak can reach a height of 20,000 RFU and still fall within the ideal of 10,000 RFU per allele. This is still well below the saturation threshold for the ABI 3500xl (which is typically around 30,000 RFU). However, Fuji et al. [31] found that pull-up occurs at approximately 1% of the parent peak height, meaning that for peaks in the 10,000–20,000 RFU range pull-up peaks of around 100–200 RFU might be expected. This is in the range where forensic laboratories typically set their analytical threshold (50 RFU to 250 RFU from author personal experience) and therefore is at the point when these pull-up peaks will start to affect the interpretation of profiles. Ultimately, the revised profile quality score accurately reflects meaningful differences in quality, rather than a narrowly defined ideal.

The revised metric determines the profile quality score f(P) for a DNA profile P with peak heights of x_1_, …, x_n_ using

(1) $$f\left(P\right)=K_{0}[\log_{10}\left[p\left({\bar{x}}_{adj}|\mu_{a},\sigma_{a}\right)\right]-C+K_{1}\log_{10}\left[p\left(c_{v}|\lambda \right)\right]+K_{2}n_{a}+K_{3}t$$

where

$\bar{x}=\frac{1}{N}\sum_{i=1}^{n}x_{i}$ is the mean of all observed allelic peaks.
${\bar{x}}_{adj}$ is the transformed version of the observed mean peak height ($\bar{x}$).
  $${\bar{x}}_{adj}=\left\{\begin{array}{ll} \bar{x} & \bar{x}<5000\\ \mu_{a} & 5000\leq \bar{x}\leq 10000\\ \bar{x}-5000 & \bar{x}>10000 \end{array}\right.$$

**Figure 1:**
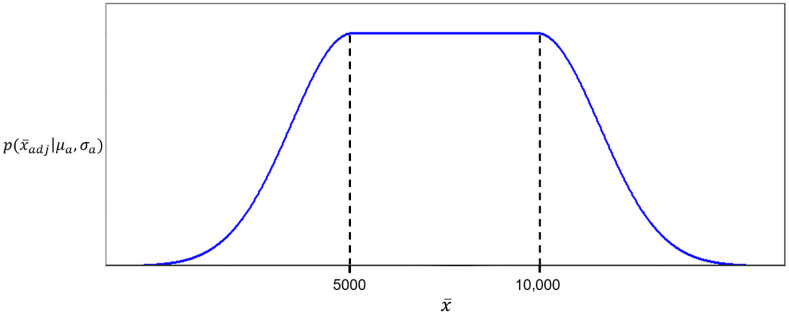
A graphical illustration of the peak height penalty function. Average peak heights below 5000 and above 10,000 RFU, which represent the upper and lower acceptable limits (U and L), experience a gradually increasing penalty, while values between 5000 and 10,000 RFU are treated equally and retain the highest weight (flat plateau), reflecting no penalty.

Graphically, the penalty applied is shown in Figure 1 below. However, this equation could be generalised to between any acceptable limits (lower, L, and upper, U) by

$${\bar{x}}_{adj}=\left\{\begin{array}{ll} \bar{x} & \bar{x}<L\\ \mu_{a} & L\leq \bar{x}\leq U\\ \bar{x}-T+L & \bar{x}>U \end{array}\right.$$

$p\left({\bar{x}}_{adj}|\mu_{a},\sigma_{a}\right)∼N\left(\mu_{a},\sigma_{a}\right)$ is the probability density function at the point ${\bar{x}}_{adj}$ for the distribution with mean $\mu_{a}$ (i.e., the ideal or reference mean value) and standard deviation $\sigma_{a}$ (i.e., the value for weighting variations from the ideal).

${C=\log}_{10}\left[p\left(\mu_{a}|\mu_{a},\sigma_{a}\right)\right]+{log}_{10}\left(\lambda \right)$ is the constant offset to ensure an ideal peak height achieved a score of 0.

$p\left(c_{v}|\lambda \right)∼\exp\left(\lambda \right)$ is the exponential distribution at the point $c_{v}$ with coefficient λ.

$c_{v}=\frac{\sqrt{\frac{1}{N}\sum_{x=1}^{n}{\left(x_{i}-\bar{x}\right)}^{2}}}{\bar{x}}$ is the coefficient of variation (COV) in the peaks.

$n_{a}$ is the number of observed artefact peaks.

*t* is the time (in minutes) the PCR program takes to complete.

*K* parameters weigh the importance of each element of the score.

With reference to Equation (1), we used values $K_{0}=1$, $\mu_{a}=5000$, T = 10,000, $\sigma_{a}=1000$, λ=1 and $K_{2}=-2$. The choice of parameter values for the target peak height ($\mu_{a}$), upper threshold peak height (T) and standard deviation ($\sigma_{a}$) was based on the previous work and personal experience of the authors [22]. In this study the time component, $K_{3}$, was set to 0 as total PCR runtime was not relevant. The two main quality components (allele peak height and allele peak height variation) were approximately equally weighted, while a single artefact in the data would contribute approximately equally to any one of the other components.

Likelihood ratios (LRs) were calculated for all replicates using the probabilistic genotyping software STRmix^TM^ v2.9.0 using the GlobalFiler specific settings. The reference profile of the donor was compared to the profiles generated using the various STR and qPCR kit combinations to generate a likelihood ratio (LR). These LR calculations considered the following propositions:

**H1:**  *The known donor is the source of the DNA.*

**H2:**  *An unknown, unrelated individual is the source of the DNA.*

The sub-source LRs were calculated and reported based on the Australian Caucasian population [32], as this was the known racial background to which the donor identified. In Australia the National Criminal Investigation DNA Database (NCIDD) defines an informative DNA profile as a profile that contains 12 or more alleles from a single contributor, thus profiles containing 12 or more alleles from the donor were deemed informative. A robust linear mixed-effects model [33] was used to analyse the effect of humic acid concentration and PCR setup on average peak heights. Any significant variation in the number of observed alleles, percent allele loss and quality scores were determined using Welch’s ANOVA and post hoc Games-Howell tests, while significant variation in the sub-source LRs between the three setups for each STR kit was identified using Kruskal–Wallis and post hoc Dunn’s tests. All statistical analyses were performed in R [34] and *p*-values below 0.05 were considered significant.

## 3. Results & Discussion

### 3.1. Standard GlobalFiler Setups

All five replicates generated full DNA profiles from the two lowest quantities of humic acid (50 ng and 100 ng) all of which produced sub-source LRs that indicate high evidentiary value (Table 1). The profiles produced in the presence of 50 ng of humic acid had average peak heights that were comparable to the uninhibited (no humic acid) profiles, with values of 5129 and 5170 RFU, respectively (Table 2). However, despite generating full profiles, the samples in the presence of 100 ng of humic acid had lower peak heights than the uninhibited profiles, with an average height of 4149 RFU (Table 2). The 50 ng and 200 ng humic acid profiles contained greater inter- and intra- locus imbalance, observed as larger COV values, and contained more artefacts than the uninhibited profiles (Table 2). This increase in artefacts was expected, as humic acid is known to inhibit DNA polymerase activity [17,27,35,36]. As a result, off-target PCR products are observed within the resulting DNA profiles. Due to their broad and short morphologies, which are characteristic of inhibition, these artefactual peaks were easily identifiable in the profiles and did not cause issues during deconvolution or interpretation. As expected, the addition of 50 ng and 100 ng of humic acid to the reactions did result in lower average profile quality scores (−21.25 and −19.61, respectively) than the uninhibited profiles (−8.82). However, after standardisation to the profiles with no humic acid added, a variation in profile quality was found to be statistically insignificant at both concentrations. Notably, the variation between profile features and overall quality between the 50 ng and 100 ng profiles was minimal, which suggests that both concentrations of humic acid exert a comparable inhibitory effect on a standard GlobalFiler setup with an ideal amount of template DNA. These findings also indicate that the DNA polymerase within the GlobalFiler reagents exhibits tolerance to humic acid at these lower concentrations.

In the profiles generated using 200 ng of humic acid, a significant decline was observed in the number of alleles recovered, with three samples producing a full profile and two samples generating partial profiles–one of which contained only six alleles (Table 1; Supplementary Table S2). This led to substantial variation in profile quality scores, which ranged from −12.07 to −47.83 (Supplementary Table S1). The variation in alleles recovered and overall quality between replicates at this concentration of humic acid may be the result of a differences in the amount or distribution of inhibitor present when sampling from the stock solution, especially because humic acid is not highly soluble and can be heterogenous in solution [37]. However, another potential contributing factor could be the timing of inhibitor activity during the PCR. If inhibition occurs early during amplification, DNA polymerase activity is restricted from the outset, leading to poor initial amplification. As a result, the poorly amplified STR targets serve as templates in subsequent cycles with the effect of compounding the amplification inefficiency in the early cycles which significantly reduces profile informativeness. Alternately, if the inhibitor takes a few cycles to act on the polymerase, early amplification can proceed efficiently and allow good quality target amplicons to be produced before inhibition takes full effect resulting in informative profiles being generated. While this hypothesis is consistent with the exponential dynamic of PCR and known inhibitor mechanisms [6,17,38], it remains speculative. Additional research would be required to determine if the timing of inhibitor action meaningfully contributes to variability in profile quality alongside the more likely influence of humic acid’s heterogenous and poorly soluble nature.

The profiles generated with 200 ng of humic acid had a significantly lower average peak height of 872 RFU compared to the uninhibited profiles (*p* = 0.048) (Table 2). Increased inter- and intra- locus imbalance was present across the 200 ng inhibited profiles, observed through the high average COV value of 1.64 (Table 2), but this was expected. Additionally, an increased number of artefacts characteristic of polymerase inhibition were present in these profiles; however, the number of artefacts in the 200 ng profiles was comparable to those present in the 50 ng and 100 ng profiles (Table 2). As expected, all these profiles’ features contributed to an overall lower average profile quality score of −26.92 (Table 2) when compared to the uninhibited. Despite this, the variation in profile quality between the uninhibited and 200 ng of humic acid was not found to be statistically significant. While the LRs generated from these profiles ranged from moderate to extremely high evidentiary value (Table 1), the average LR for the 200 ng profiles was not found to be statistically significantly lower than those of the uninhibited profiles. However, this was expected given the amount of template DNA used in this study (500 pg) and it would be expected that the effect of 200 ng of humic acid on profile quality would be more substantial if suboptimal amounts of DNA were used.

The 300 ng samples all produced partial profiles with two samples not recovering enough alleles to meet the upload requirements for the Australian NCIDD (Supplementary Table S2). These profiles exhibited an average peak height of 403 RFU (Table 2), which was found to be statistically significantly lower than the uninhibited profiles (*p* = 0.035). The 300 ng profiles also contained significantly fewer alleles than the uninhibited profiles, with an average percent loss of approximately 58% (22 alleles) (Table 1). This variation in total observed alleles when compared to the uninhibited profiles was also found to be statistically significant (*p* = 0.009). Increased inter- and intra- locus imbalance was also noted across the 300 ng profiles in comparison to the uninhibited profiles, as reflected by the average COV value of 2.16 (Table 2). Additionally, there was a number of artefacts present in the 300 ng profiles, as reflected in the artefact penalty (Table 2), however, there were not substantially more artefacts present than in the 200 ng profiles. Lastly, the evidentiary value of these profiles was lower than the uninhibited, as shown by the average sub-source LR of 2.91 × 10^18^ (Table 1). This variation in LRs was expected given the poor quality of the 300 ng profiles and was found to be statistically significantly lower when compared to the uninhibited profiles (*p* = 0.008).

### 3.2. Combined GlobalFiler & Investigator Quantiplex Pro Setup

Full DNA profiles were obtained from all replicates containing humic acid concentrations between 0 ng (uninhibited) and 200 ng using the combined GlobalFiler and Investigator Quantiplex Pro setup (Table 3). The combined setups using 50, 100 and 200 ng of humic acid all produced profiles with comparable average peak heights to the uninhibited (0 ng) profiles, with values of 7049, 5551, 6361 and 7438 RFU, respectively (Table 4). A notable and proportionate reduction in average peak height was observed as the concentration of inhibitor increased to 300 ng/μL, with the slight deviation for the 200 ng/μL samples likely reflecting the solubility and early/late inhibitory effects discussed earlier. Notably the COV scores show little change across the three lowest humic acid concentrations (50, 100 and 200 ng), suggesting that the expected inhibitory effects, such as the preferential loss of peaks at larger loci and increased heterozygous imbalance, were effectively minimised by the use of the combined GlobalFiler and Investigator Quantiplex Pro reagents.

This is further highlighted by the average profile quality score of −11.83 for the 50 ng profiles (Table 4) which is comparable to the uninhibited profiles, which had an average score of −10.04 (Table 4). The number of artefacts present in the 100 ng and 200 ng profiles was greater than those observed in the 50 ng profiles; however, the number of artefacts observed was still consistent with those expected of inhibitors at this concentration. As a result of the increased number of artefacts present in the 100 ng and 200 ng profiles, the overall quality of theses profiles was substantially lower than the uninhibited profiles, with average quality scores of −18.85 and −17.42, respectively (Table 4). However, as stated previously, the presence of artefactual peaks was not detrimental to profile deconvolution or interpretation, as the peaks were easily identifiable due to their unusual morphology. Importantly, the variation in profile features and quality between the 50, 100 and 200 ng profiles was not found to have a statistically significant impact on the evidentiary value of the profiles, as indicated by the consistently large sub-source LRs for all samples at these concentrations (Table 3).

The 300 ng of humic acid combined setup profiles exhibited an average allele loss of 30% (11 alleles) (Table 3). However, of the five replicates, two produced full profiles, one was near complete (34/37 alleles), one was a partial profile (15/37 alleles), and one was a very poor profile (8/37 alleles) that did not meet the requirements for upload to the Australian NCIDD (Supplementary Table S4). As stated previously, this variation highlights the inherent range in profile qualities observed at higher inhibitor concentrations relating to the stochastic nature of inhibition in the early cycles. The average peak height in the 300 ng profiles was 3082 RFU, which was approximately 4000 RFU lower than the profiles with no inhibitor (Table 4), but statistically significantly higher than the standard GlobalFiler profiles at the same inhibitor concentration (*p* < 0.01). Additionally, as expected at the highest concentration of inhibitor, increased artefact presence and inter- and intra-locus balance was noted in the 300 ng profiles; this was indicated by the average artefact and COV penalties of −16.00 and −3.02, respectively, that were imparted during scoring (Table 4). Each of these features culminated in an average profile quality score of −27.89 for these 300 ng profiles (Table 4). Despite this, the presence of 300 ng of humic acid was not found to have a significant impact on the evidentiary value of the profiles generated, with an average sub-source LR of 4.65 × 10^25^ for these samples (Table 3). However, it is important to note that there was substantial variation in the LR values calculated for each for the 300 ng samples as a result of the range in profile quality noted (Table 3; Supplementary Tables S3 and S4).

Standardising all profile quality scores against the standard GlobalFiler setup with no inhibitor enables direct comparison between inhibited and uninhibited samples relative to an ideal baseline. This is essential to assess whether altering the PCR setup (such as combining endpoint and qPCR reagents) can overcome inhibition and recover profile quality to a level comparable to the standard uninhibited profiles. Notably, when inhibited profiles generated using the combined setup were standardised in this way, their quality scores were not statistically significantly different from those of the standard GlobalFiler profiles with no inhibitor present. Thus, indicating that the modified setup successfully minimised the effects of inhibition during amplification.

### 3.3. Standard Endpoint PCR with Additional Taq Polymerase and Buffer

Full DNA profiles were only obtained from all replicates containing no humic acid (uninhibited) using the GlobalFiler with additional master mix setup (Table 5). These profiles had an average peak height of 6467 RFU (Table 6). The GlobalFiler with additional master mix setups using 50, 100 and 200 ng of humic acid had similar average peak heights of 4535, 4808 and 4716 RFU, respectively (Table 6). While these peak heights were notably lower than those of the uninhibited profiles from the same reaction setup, the variation was not statistically significant. As with the combined setup, the COV scores showed little change across the three lowest humic acid concentrations (50, 100 and 200 ng in Table 6), further supporting the hypothesis that additional buffer and polymerase helped minimise inhibitory effects.

When 50 ng of humic acid was added, profiles exhibited an average allele loss of 15% (5 alleles) (Table 5), although all five replicates met NCIDD upload requirements, with three replicates producing full profiles, one a near complete profile (32/37 alleles), and one partial profile (15/37 alleles) (Supplementary Table S6). Profiles with 100 ng of humic acid showed an average allele loss of approximately 3% (1 allele) (Table 5), with two replicates generating full profiles and three producing near complete profiles (35/37 or 36/37 alleles) (Supplementary Table S6). In contrast, profiles with 200 ng of humic acid showed an average allele loss of approximately 11% (4 alleles) (Table 5); three replicates produced full profiles, one a near complete profile (32/37 alleles) and one a partial profile 22/37 alleles (Supplementary Table S6).

Across the lowest three humic acid concentrations, the resulting profiles also contained fewer artefacts than expected. This was reflected in the relatively low artefact penalties, with average penalties of −0.40, −4.40 and −3.20 for the 50, 100 and 200 ng of humic acid profiles, respectively (Table 6). Although the 100 ng profiles had a slightly higher number of artefacts compared to the 50 and 200 ng profiles, the overall presence of artefacts was lower than expected at this inhibitor concentration. This was further highlighted by the average profile quality scores of −10.50, −13.04 and −12.39 for the 50, 100 and 200 ng profiles, respectively; these were comparable in quality to the profiles with no humic acid (−9.75) produced with the same setup (Table 6). As with previous setups, the presence of artefactual peaks in these profiles was not detrimental to deconvolution or interpretation as they were easily identifiable due to their unusual morphology. Notably, the differences in profile features and quality observed between the 50, 100 and 200 ng profiles did not substantially affect the evidentiary strength of the resulting profiles, with all samples at these concentrations producing sub-source LRs that provided extremely strong support for the inclusion of the donor (Table 5; Supplementary Table S6).

The 300 ng of humic acid profiles produced using the GlobalFiler setup with additional master mix exhibited an average allele loss of 33% (12 alleles) (Table 5). Of the five replicates, one failed to produce a profile, while the remaining four produced two near complete profiles (30/37 alleles) and two partial profiles (24/37 and 15/37 alleles) (Supplementary Table S6). The variation in these profiles again highlights the inherent variation in profile qualities observed at higher inhibitor concentrations. The average peak height in the 300 ng profiles was 2797 RFU, which was approximately 3500 RFU lower than the profiles with no inhibitor (Table 5). As expected, increased inter- and intra-locus balance was also observed, reflected by an average COV of 1.34 (Table 6). Interestingly, despite the concentration of inhibitor, the number of artefacts present in the 300 ng profiles was substantially lower than expected. The 300 ng profiles showed an average artefact penalty of −2.00, which was lower than the penalties imparted on the 100 ng and 200 ng profiles (−4.40 and −3.20, respectively) (Table 6). When overall profile quality of the 300 ng profiles was assessed, the increased imbalance in the profiles was offset by the reduced artefact penalties, resulting in an average profile quality score of −12.96 (Table 6). The presence of 300 ng of humic acid was not found to have a significant impact on the evidentiary value of the profiles generated, with an average sub-source LR of 3.60 × 1020 for these samples (Table 5). However, it is important to note that there was substantial variation in the LR values calculated for each for the 300 ng samples as a result of the range in profile quality noted (Table 5; Supplementary Tables S5 and S6).

When profile quality scores were standardised against the standard GlobalFiler setup with no inhibitor, the profiles generated using the GlobalFiler with additional buffer and polymerase were not found to be significantly different in quality to the standard uninhibited GlobalFiler profiles. Notably, the profile produced using the modified GlobalFiler setup with 300 ng of humic acid were statistically significantly better quality than the standard 300 ng profiles (*p* = 0.002). This indicates that the addition of extra buffer and polymerase enhanced amplification efficiency and improve profile quality, even at the highest level of inhibition.

### 3.4. Standard Endpoint PCR with Additional qPCR Taq Polymerase and Buffer

Full profiles were obtained from all replicates containing no humic acid (uninhibited), 50 ng, 100 ng and 200 ng of humic acid using the GlobalFiler with Investigator Quantiplex Pro reaction mix setup (Table 7). Profiles generated with 50, 100 and 200 ng of humic acid exhibited comparable or greater average peak heights than the uninhibited profiles, with values of 8456, 7727, 7624 and 7023, respectively (Table 8). Although a reduction in average peak height was observed between the 50 ng and 100 ng profiles, the difference between the 100 ng and 200 ng profiles was negligible (Table 8). None of this variation was found to be statistically significant. The expected proportional decrease in peak heights as inhibitor concentration increased (as noted in the standard GlobalFiler results) was not observed in any of these profiles, suggesting that the reaction setup can effectively mitigate the effects of humic acid inhibition on allelic peak heights. Notably, COV scores showed little variation across the three lowest humic acid concentrations (50, 100 and 200 ng), and indicated less inter- and intra-locus imbalance compared to the uninhibited profiles (Table 8). These results provide strong additional support for the hypothesis that the preferential loss of peaks at larger loci and the heterozygous imbalance typically associated with inhibition were effectively minimised by supplementing the GlobalFiler reaction with Investigator Quantiplex Pro reaction mix (containing a different polymerase and buffer).

Additionally, the 50, 100 and 200 ng profiles produced using this setup contained substantially fewer artefactual peaks than expected. This is reflected in the very low artefact penalties imparted on these profiles that were comparable to the uninhibited, with the 0, 50, 100 and 200 ng profiles having average penalties of 0, 0, −0.80 and −0.80, respectively (Table 8). This consistency in profile quality is further highlighted by the profile quality scores for the uninhibited, 50, 100 and 200 ng profiles, which had averages of −8.41, −8.00, −8.83 and −8.86, respectively (Table 8). As expected, this consistency in profile quality was also evident in the sub-source LRs of the profiles at these concentrations, with all samples providing extremely strong support for inclusion of the donor (Table 7; Supplementary Table S8).

The profiles produced with 300 ng of humic acid exhibited an average allele loss of approximately 5% (2 alleles) (Table 7). Interestingly, all five replicates met the requirements for uploading to the NCIDD, with two producing full profiles and the other three producing almost complete profiles (30/37 alleles, 35/37 alleles and 36/37 alleles) (Supplementary Table S8). The variations in profile qualities observed at higher inhibitor concentrations were expected; however, in comparison to the variation observed in other setups in this study, the variation between replicates using this setup was notably lower and not found to be statistically significant. As expected, additional inter- and intra-locus imbalance was observed in the 300 ng profiles compared to the 50, 100 and 200 ng profiles (Table 8); however, the average COV of 0.85 for the 300 ng profiles was substantially lower than anticipated. Similarly, the expected increase in artefact presence between the 100, 200 and 300 ng profiles was not observed, with identical average artefact penalties of −0.80 (Table 8) recorded for each concentration. The addition of 300 ng of humic acid also did not statistically significantly impact the evidentiary value of the resulting profiles, with an average sub-source LR of 9.25 × 1025 providing extremely strong support for the inclusion of the donor (Table 7). Although some variation in LR values was observed between the 300 ng profiles, it was considerably less than that seen with the other setups tested in this study (Table 7; Supplementary Table S8).

When profile quality scores were standardised against the standard GlobalFiler setup with no inhibitor, the profiles generated using the GlobalFiler setup supplemented with Investigator Quantiplex Pro buffer and polymerase were not found to be significantly different in quality to the standard uninhibited GlobalFiler profiles. Additionally, the supplemented profiles generated in the presence of 300 ng of humic acid were found to be statistically significantly better in quality than standard 300 ng profiles (*p* = 0.002). Interestingly, the profiles generated using the GlobalFiler setup supplemented with Investigator Quantiplex Pro buffer and polymerase only (no primer mix) in the presence of 100 and 200 ng of humic acid were found to be statistically significantly better in quality than the profiles produced using the combined GlobalFiler and Investigator Quantiplex Pro setup (with both reaction and primer mix) (*p* = 0.016 and 0.025, respectively). This result demonstrates that the addition of an alternative commercial buffer and polymerase was able to enhance amplification and maintain high profile quality, even under substantial inhibitory pressure. Notably, these findings suggest that the improved resistance to inhibition is primarily due to the addition of extra buffer and polymerase components (from the reaction mix), rather than the full qPCR mixture (reaction mix + primer mix). This is further supported by the consistent peak heights observed across conditions containing GlobalFiler with either additional GlobalFiler master mix or Investigator Quantiplex Pro. The key difference lies in the introduction of artefacts when the primer mix is included, likely due to increased primer-primer interactions rather than a loss of inhibition resistance. This indicates that the enhancement in profile quality under inhibited conditions can be attributed to the increased concentration of reaction mix components alone (polymerase and buffer), while the inclusion of additional primers does not negate this benefit it also introduces artefacts that will lower the overall profile quality score. Therefore, increasing the concentration of the buffer and polymerase components alone is sufficient to improve profile quality under inhibited conditions.

### 3.5. Comparison Between Standard and Modified PCR Setups

All three modified PCR setups exhibited notable improvements in performance compared to the standard GlobalFiler setup: there was an increase in the number of alleles amplified, average peak heights were increased, LRs were greater and in some cases inter-replicate variation was reduced.

**Figure 2:**
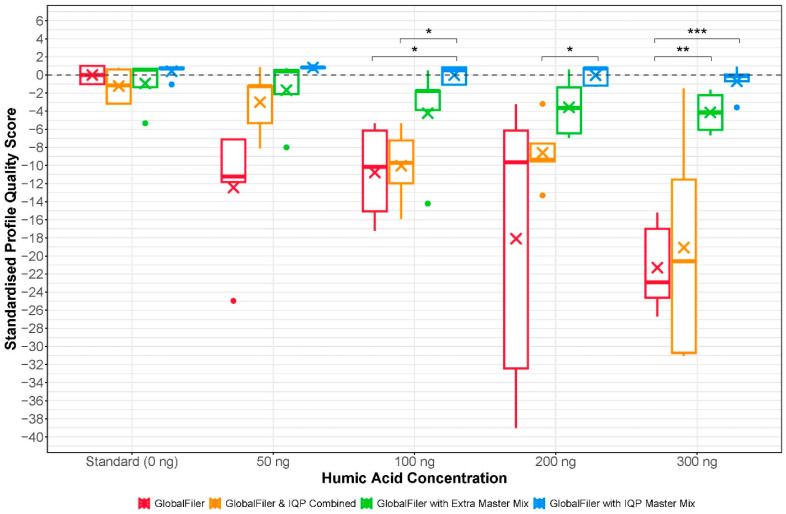
Boxplots demonstrating the spread in standardised total profile quality scores across the DNA profiles generated using either the GlobalFiler setup (red), the combined GlobalFiler and Investigator Quantiplex Pro combined setup (orange), the GlobalFiler setup with extra master mix (green) or the GlobalFiler setup with extra Investigator Quantiplex Pro master mix (blue). For each of the four setups trialled the profiles generated from samples containing the same amount of humic acid were compared and significant differences in profile quality scores are indicated by asterisks (*). Where one asterisk denotes a *p*-value < 0.05, two asterisks denote a *p*-value < 0.01 and three asterisks denote a *p*-value < 0.001. The means are indicated by an X and for all protocols *n* = 5. The total number of profiles analysed was 100.

Under uninhibited or low inhibitory conditions (0 or 50 ng of humic acid) the profile quality scores for the GlobalFiler only setup was comparable, or slightly worse, than all the modified setups (Figure 2). The variation noted between setups at these concentrations was primarily due to the presence of larger peak heights in the combined setup profiles (Table 2, Table 4, Table 6 and Table 8). Importantly, the average peak heights in the profiles produced using the GlobalFiler setups supplemented with additional polymerase and/or buffer contained significantly larger peaks than the standard GlobalFiler setup at these inhibitor concentrations (all *p* < 0.001). Despite the improved amplification observed there was no significant decrease in profile quality observed between the modified setups and the standard GlobalFiler setup in the presence of 0 or 50 ng of humic acid (Figure 2). This indicates that any of these setups could be used without being detrimental to profile quality at low concentrations of inhibitor.

However, as the concentration of inhibitor increased (100, 200 and 300 ng) the quality of the modified setups became comparable or significantly better than their GlobalFiler only (standard) counterparts (Figure 2). The improved profile quality of all three modified setups came from improved allele recovery, improved inter- and intra-locus imbalance and reduced artefact presence (Table 1, Table 2, Table 3, Table 4, Table 5, Table 6, Table 7 and Table 8). Additionally, the average peak heights in the profiles generated using the standard GlobalFiler setup were statistically significantly lower than all profiles generated using the modified setups at 200 and 300 ng of humic acid (all *p* < 0.001) (Table 2, Table 4, Table 6 and Table 8). As a result, the standard GlobalFiler profiles obtained substantially harsher penalties during scoring and had much lower profile quality scores than all three modified setups. When the 100, 200 and 300 ng profiles were standardised against the standard GlobalFiler (0 ng humic acid) profiles, they were found to not be significantly different in quality to a standard uninhibited GlobalFiler profiles. Importantly, standardisation also indicated that the profiles produced using the GlobalFiler setups with additional polymerase and buffer (either the extra GlobalFiler master mix or Investigator Quantiplex Pro reaction mix) were of significantly greater quality than the standard GlobalFiler setup at the same concentration of inhibitor.

The gradual and substantial decrease in profile quality noted as the concentration of inhibitor increased in the standard GlobalFiler setup was expected. As previously stated, the gradual decrease in profile quality observed using this setup came from a gradual decrease in average peak height, poorer allele recovery and a gradual increase in profile inter- and intra-locus imbalance. However, when the modified setups were used to amplify the inhibited samples, this decrease in profile quality is almost entirely eliminated. This was noted in both the combined GlobalFiler and Investigator Quantiplex Pro setup and the GlobalFiler with extra master mix setup, relative to the standard GlobalFiler setup (Figure 2). Importantly, this decrease is greatly diminished in the GlobalFiler profiles supplemented with additional master mix in comparison to the profiles from the standard and combined GlobalFiler and Investigator Quantiplex Pro setups (Figure 2).

The levels and types of inhibition-related artefacts were comparable between the setups at each concentration of humic acid, indicating that modifying the reaction setup did not introduce additional or exacerbate existing inhibitory effects. The presence and number of artefactual peaks observed increased with inhibitor concentration in the profiles produced by most of the setups. However, in the case of the profiles generated using a GlobalFiler reaction supplemented with additional Investigator Quantiplex Pro polymerase and buffer, there was no notable increase in inhibition-related artefacts across concentrations. This indicates that the use of a modified reaction comprising two different DNA polymerases and commercial buffers can improve peak heights and profile balance, as well as minimise the off-target amplification that is characteristic of inhibited samples.

The overall conservation of profile quality at all concentrations of humic acid with the two supplemented GlobalFiler reactions suggests increased inhibitor tolerance using these setups. The data indicates this is likely due to the presence of additional DNA polymerase and/or buffer in the reaction vessel. Doubling the polymerase present in the reaction vessel by either adding more of the same polymerase (by adding more GlobalFiler master mix) or adding a second polymerase (by adding Investigator Quantiplex Pro reaction mix) may provide redundancy for the enzyme, meaning that while the inhibitor is acting to reduce efficiency of one of the polymerases, the other polymerases present are still able to amplify the STR targets efficiently. Additionally, the relative inhibitor-to-polymerase ratio present in the modified reaction setups may be further contributing to the increased profile quality at the various inhibitor concentrations. The results suggest that the use of a different polymerase, which may possess greater resistance to inhibition due to its source, structure or reaction formulation, contributed to the improved amplification observed in the profiles produced using the GlobalFiler supplemented with Investigator Quantiplex Pro reaction mix (Figure 2).

Interestingly, the results also indicate that the inclusion of qPCR primer (from the primer mix) does not markedly improve or diminish profile quality compared to the standard setup (Figure 2). While this suggests the addition of qPCR primer mix is not detrimental, it may not be necessary unless real-time monitoring of the amplification process is desired. For applications where monitoring is not required (i.e., in current operational DNA profiling workflows), simply supplementing a standard setup with additional polymerase and buffer appears sufficient. However, if real-time monitoring of amplification is of interest (i.e., for a smart PCR system as described in [28,30]), further optimisation of the combined endpoint PCR and qPCR setup, such as altering the ratio of qPCR reaction mix to primer mix, warrants investigation.

Furthermore, increasing the buffer components may also be providing increased ionic strength that is enhancing amplification beyond that of the standard GlobalFiler setup. While the exact components of each buffer are unknown, increasing the amount of magnesium and potassium ions (Mg^2+^ and K^+^) by combining the two kits may help promote amplification by stabilising the DNA template-primer complex, catalyse bond formation between primers and dNTPs [9,39,40] and promoting primer annealing [39,41]. Given that all three modified setups contained a greater amount of buffer than a standard GlobalFiler reaction, it is possible that the additional buffer also contributed to the improved amplification at all inhibitor concentrations, as reflected in the consistently higher profile qualities compared to the standard setup (Figure 2). However, because the supplemented setups demonstrated similar performance across the range of inhibitor concentrations tested, the data does not allow for clear differentiation between the benefits of simply increasing the buffer from the same kit (as in the GlobalFiler with additional GlobalFiler master mix) versus combining buffers from two different commercial kits (as in the GlobalFiler with Investigator Quantiplex Pro reaction mix).

The proprietary nature of these commercial kits, and the fact the buffer and polymerase are pre-mixed, makes it challenging to determine whether the improve inhibitor tolerance in the modified setups stems from the buffer, the polymerase or their combination. Nonetheless, the results of this study demonstrate that supplementing reactions in this way is a promising strategy to mitigate inhibition.

## 4. Conclusions

This study demonstrated that modified PCR setups outperform the standard GlobalFiler approach when generating informative DNA profiles from humic acid-inhibited samples, including at the highest concentration tested. The modified PCR setups generally produced higher-quality profiles with improved allele recovery and overall balance compared to the standard protocol at higher inhibitor concentrations. The findings suggest that supplementing reagents to allow double polymerase and buffer components in the reaction vessel may offer a more robust and inhibitor-tolerant reaction environment for STR amplification.

Beyond demonstrating the efficacy of a modified PCR setup to overcome a longstanding challenge when profiling inhibited samples, this study also provides a comprehensive dataset characterising the impact of increasing humic acid concentrations on DNA profile quality from an ideal amount of high-quality DNA template. Thus, this provides valuable insights that can allow further optimisation of profiling methodologies for inhibited samples in the future.

The minimised presence of inhibition-associated artefacts across all modified PCR setups highlights the opportunity for future optimisation of the PCR process itself, particularly in reducing off-target amplification under inhibited conditions. Importantly, humic acid is known to inhibit PCR through several mechanisms, including polymerase interference, DNA binding and fluorescence quenching, which are shared by many other inhibitors common in forensic casework samples.

It is acknowledged that this study was conducted using DNA from a single donor, and while the results are a strong proof of concept, further work using multiple donors will be important to confirm the broader applicability of these findings. The models in STRmix are robust to the effects of inhibition to a certain degree; however, single-source profiles (which typically only have a single reasonable genotypic explanation at each locus) extended this robustness. Mixed samples, where there are multiple combinations of explanatory genotypes, may demonstrate different limits to acceptable inhibition than single-source profiles. Although the same trend of the combined buffer chemistries performing better than GlobalFiler alone is expected on mixed profiles, the exact effects on mixture deconvolution would provide information on the practical application limits to typical mixed casework samples. This is one example of additional work that would be required before the technique could be applied in an operational laboratory. Other considerations would include the way in which the method could fit within a typical operational workflow, whether the combined kit chemistries require additional modelling for analysis in probabilistic genotyping systems like STRmix, and the full validation requirements to maintain ISO accreditation.

The success of modified approaches to amplification may extend beyond humic acid to a broader range of challenging sample types. However, inhibitor-specific empirical studies would be required to confirm this. Ultimately, these findings highlight the potential of modified PCR workflows to improve DNA profiling outcomes from inhibited samples and present a promising approach to overcome a common challenge in forensic science.

## Figures and Tables

**Table 1:** The average number of observed alleles, average percent allele loss and average sub- source likelihood ratios for the STR profiles produced from 500 pg of DNA template with the standard GlobalFiler setup and various concentrations of humic acid added. In brackets below, each average sub-source likelihood ratio is the range of likelihood ratios generated using that PCR program. For each program, *n* = 5. For all profiles, the expected number of donor alleles was 37.

Humic Acid (ng/µL)	Average Number of Observed Alleles	Average Percent Allele Loss (%)	Average Sub-Source Likelihood Ratio
0 (Uninhibited)	37	0.00	2.26 × 10^26^ (2.26 × 10^26^–2.26 × 10^26^)
50	37	0.00	2.23 × 10^26^ (2.12 × 10^26^–2.26 × 10^26^)
100	37	0.00	1.16 × 10^26^ (1.55 × 10^25^–2.26 × 10^26^)
200	26	29.19	1.35 × 10^26^ (8.16 × 10^2^–2.26 × 10^26^)
300	16	57.84	2.91 × 10^18^ (1.32 × 10^1^–1.45 × 10^19^)

**Table 2:** The breakdown of the average profile quality scores assigned to the STR profiles produced from 500 pg of DNA template with the standard GlobalFiler setup and various concentrations of humic acid added. For each concentration of humic acid, *n* = 5.

Humic Acid (ng/µL)	Average Peak Heights (RFU)			Average Penalties			Average Profile Quality Score
	Mean	Std. Dev.	COV	Peak Height	COV	Artefacts	
0	5170	2435	0.47	−3.60	−1.02	−0.80	−8.82
50	5129	2924	0.66	−4.42	−1.43	−12.00	−21.25
100	4149	2992	0.76	−3.76	−1.65	−10.80	−19.61
200	872	932	1.64	−7.17	−3.55	−12.80	−26.92
300	403	674	2.16	−8.02	−4.69	−14.00	−30.11

**Table 3:** The average number of observed alleles, average percent allele loss and average sub- source likelihood ratios for the STR profiles produced from 500 pg of DNA template with the combined qPCR and endpoint PCR setup and various concentrations of humic acid added. In brackets below, each average sub-source likelihood ratio is the range of likelihood ratios generated using that PCR program. For each program, *n* = 5. For all profiles, the expected number of donor alleles was 37.

Humic Acid (ng/µL)	Average Number of Observed Alleles	Average Percent Allele Loss (%)	Average Sub-Source Likelihood Ratio
0 (Uninhibited)	37	0.00	1.92 × 10^26^ (5.62 × 10^25^–2.26 × 10^26^)
50	37	0.00	7.94 × 10^25^ (2.36 × 10^24^–2.26 × 10^26^)
100	37	0.00	1.92 × 10^26^ (5.62 × 10^25^–2.62 × 10^26^)
200	37	0.00	1.92 × 10^26^ (5.62 × 10^25^–2.62 × 10^26^)
300	26	29.19	4.65 × 10^25^ (1.30 × 10^5^–2.62 × 10^26^)

**Table 4:** The breakdown of the average profile quality scores assigned to the STR profiles produced from 500 pg of DNA template with the combined qPCR and endpoint PCR setup and various concentrations of humic acid added. For each concentration of humic acid, *n* = 5.

Humic Acid (ng/µL)	Average Peak Heights (RFU)			Average Penalties			Average Profile Quality Score
	Mean	Std. Dev.	COV	Peak Height	COV	Artefacts	
0	7438	4252	0.57	−3.40	−1.24	−2.00	−10.04
50	7049	4453	0.66	−3.40	−1.43	−3.60	−11.83
100	5551	3960	0.74	−3.45	−1.60	−10.40	−18.85
200	6361	4070	0.65	−3.41	−1.41	−9.20	−17.42
300	3082	2531	1.39	−5.48	−3.02	−16.00	−27.89

**Table 5:** The average number of observed alleles, average percent allele loss and average sub- source likelihood ratios for the STR profiles produced from 500 pg of DNA template with the GlobalFiler with additional polymerase and buffer setup and various concentrations of humic acid added. In brackets below, each average sub-source likelihood ratio is the range of likelihood ratios generated using that PCR program. For each program, *n* = 5. For all profiles, the expected number of donor alleles was 37.

Humic Acid (ng/µL)	Average Number of Observed Alleles	Average Percent Allele Loss (%)	Average Sub-Source Likelihood Ratio
0 (Uninhibited)	37	0.00	2.26 × 10^26^ (2.26 × 10^26^–2.26 × 10^26^)
50	32	14.59	1.36 × 10^26^ (2.64 × 10^9^–2.26 × 10^26^)
100	36	2.71	9.27 × 10^25^ (4.10 × 10^23^–2.62 × 10^26^)
200	33	10.81	1.36 × 10^26^ (5.19 × 10^13^–2.62 × 10^26^)
300	25	33.11	3.60 × 10^20^ (6.83 × 10^9^–7.21 × 10^20^)

**Table 6:** The breakdown of the average profile quality scores assigned to the STR profiles produced from 500 pg of DNA template with the GlobalFiler with additional polymerase and buffer setup and various concentrations of humic acid added. For each concentration of humic acid, *n* = 5.

Humic Acid (ng/µL)	Average Peak Heights (RFU)			Average Penalties			Average Profile Quality Score
	Mean	Std. Dev.	COV	Peak Height	COV	Artefacts	
0	6467	4025	0.62	−3.40	−1.35	−1.60	−9.75
50	4535	3362	0.99	−4.54	−2.16	−0.40	−10.50
100	4808	3962	0.83	−3.45	−1.79	−4.40	−13.04
200	4716	3796	0.90	−3.83	−1.96	−3.20	−12.39
300	2797	3505	1.34	−4.64	−2.92	−2.00	−12.96

**Table 7:** The average number of observed alleles, average percent allele loss and average sub- source likelihood ratios for the STR profiles produced from 500 pg of DNA template with the GlobalFiler with additional qPCR polymerase and buffer setup and various concentrations of humic acid added. In brackets below, each average sub-source likelihood ratio is the range of likelihood ratios generated using that PCR program. For each program, *n* = 5. For all profiles, the expected number of donor alleles was 37.

Humic Acid (ng/µL)	Average Number of Observed Alleles	Average Percent Allele Loss (%)	Average Sub-Source Likelihood Ratio
0 (Uninhibited)	37	0.00	2.25 × 10^26^ (2.23 × 10^26^–2.26 × 10^26^)
50	37	0.00	2.26 × 10^26^ (2.26 × 10^26^–2.26 × 10^26^)
100	37	0.00	2.26 × 10^26^ (2.26 × 10^26^–2.26 × 10^26^)
200	37	0.00	2.26 × 10^26^ (2.26 × 10^26^–2.26 × 10^26^)
300	35	5.41	9.25 × 10^25^ (7.21 × 10^20^–2.62 × 10^26^)

**Table 8:** The breakdown of the average profile quality scores assigned to the STR profiles produced from 500 pg of DNA template with the GlobalFiler with additional qPCR polymerase and buffer setup and various concentrations of humic acid added. For each concentration of humic acid, *n* = 5.

Humic Acid (ng/µL)	Average Peak Heights (RFU)			Average Penalties			Average Profile Quality Score
	Mean	Std. Dev.	COV	Peak Height	COV	Artefacts	
0	7023	4231	0.65	−3.60	−1.41	0.00	−8.41
50	8456	4675	0.55	−3.40	−1.20	0.00	−8.00
100	7727	4252	0.56	−3.41	−1.22	−0.80	−8.83
200	7624	4407	0.58	−3.40	−1.26	−0.80	−8.86
300	5538	4414	0.85	−3.46	−1.85	−0.80	−9.51

## Data Availability

Additional data are available in the Supplementary Materials document.

## Supplementary Materials

The following supporting information can be downloaded at: https://www.mdpi.com/article/10.3390/genes16111326/s1, Table S1: The breakdown of the profile quality scores for the STR profiles produced from 500 pg of starting material and the standard GlobalFiler setup for the five concentrations of humic acid trialled. For each humic acid concentration *n* = 5; Table S2: The number of observed alleles, percent allele loss and sub-source likelihood ratios for all STR profiles produced from 500 pg of starting material using the standard GlobalFiler setup for each humic acid concentration trialled. For all profiles the expected number of donor alleles was 37. Replicates that did not meet the requirements for upload to the National Criminal Identification Database (NCIDD) have been marked with an asterisk (*); Table S3: The breakdown of the profile quality scores for the STR profiles produced from 500 pg of starting material and the combined GlobalFiler and Investigator Quantiplex Pro setup for the five concentrations of humic acid trialled. For each humic acid concentration *n* = 5; Table S4: The number of observed alleles, percent allele loss and sub-source likelihood ratios for all STR profiles produced from 500 pg of starting material using the combined GlobalFiler and Investigator Quantiplex Pro setup for each humic acid concentration trialled. For all profiles the expected number of donor alleles was 37. Replicates that did not meet the requirements for upload to the National Criminal Identification Database (NCIDD) have been marked with an asterisk (*); Table S5: The breakdown of the profile quality scores for the STR profiles produced from 500 pg of starting material with the GlobalFiler with additional polymerase and buffer setup for the five concentrations of humic acid trialled. For each humic acid concentration *n* = 5, except 300 ng of humic acid where *n* = 4 (one replicate failed to produce any profile); Table S6: The number of observed alleles, percent allele loss and sub-source likelihood ratios for all STR profiles produced from 500 pg of starting material with the GlobalFiler with additional polymerase and buffer setup for each humic acid concentration trialled. For all profiles the expected number of donor alleles was 37. Replicates that did not meet the requirements for upload to the National Criminal Identification Database (NCIDD) have been marked with an asterisk (*); Table S7: The breakdown of the profile quality scores for the STR profiles produced from 500 pg of starting material and the GlobalFiler with additional qPCR polymerase and buffer setup for the five concentrations of humic acid trialled. For each humic acid concentration *n* = 5; Table S8: The number of observed alleles, percent allele loss and sub-source likelihood ratios for all STR profiles produced from 500 pg of starting material using the GlobalFiler with additional qPCR polymerase and buffer setup for each humic acid concentration trialled. For all profiles the expected number of donor alleles was 37. Replicates that did not meet the requirements for upload to the National Criminal Identification Database (NCIDD) have been marked with an asterisk (*).

## References

[1] Mullis K., Faloona F., Scharf S., Saiki R., Horn G., Erlich H. (1986). Specific enzymatic amplification of DNA in vitro: The polymerase chain reaction. Cold Spring Harb. Symp. Quant. Biol..

[2] Griffin A., Henry J., Kirkbride K.P., Painter B., Linacre A. (2022). A survey of the effects of common illicit drugs on forensic DNA analysis. Forensic Sci. Int..

[3] Vajpayee K., Dash H.R., Parekh P.B., Shukla R.K. (2023). PCR inhibitors and facilitators—Their role in forensic DNA analysis. Forensic Sci. Int..

[4] Hedman J., Knutsson R., Ansell R., Rådström P., Rasmusson B. (2013). Pre-PCR processing in bioterrorism preparedness: Improved diagnostic capabilities for laboratory response networks. Biosecur. Bioterror..

[5] Al-Soud W.A., Rådström P. (2001). Purification and characterization of PCR-inhibitory components in blood cells. J. Clin. Microbiol..

[6] Opel K.L., Chung D., McCord B.R. (2010). A study of PCR inhibition mechanisms using real time PCR. J. Forensic Sci..

[7] Kreader C.A. (1996). Relief of amplification inhibition in PCR with bovine serum albumin or T4 gene 32 protein. Appl. Environ. Microbiol..

[8] Wang W., Wang H.-B., Li Z.-X., Guo Z.-Y. (2006). Silicon inhibition effects on the polymerase chain reaction: A real-time detection approach. J. Biomed. Mater. Res..

[9] Bermek O., Grindley N.D., Joyce C.M. (2011). Distinct roles of the active-site Mg^2+^ ligands, Asp882 and Asp705, of DNA polymerase I (Klenow fragment) during the prechemistry conformational transitions. J. Biol. Chem..

[10] Arsenault H., Kuffel A., Daeid N.N., Gray A. (2024). Trace DNA and its persistence on various surfaces: A long term study investigating the influence of surface type and environmental conditions—Part one, metals. Forensic Sci. Int. Genet..

[11] van Oorschot R.A., Ballantyne K.N., Mitchell R.J. (2010). Forensic trace DNA: A review. Investig. Genet..

[12] Griffin A., Kirkbride K.P., Painter B., Henry J., Linacre A. (2024). A systematic approach to the analysis of illicit drugs for DNA with an overview of the problems encountered. Forensic Sci. Int..

[13] Bhoyar L., Mehar P., Chavali K. (2024). An overview of DNA degradation and its implications in forensic caseworks. Egypt. J. Forensic Sci..

[14] Combs L.G., Warren J.E., Huynh V., Castaneda J., Golden T.D., Roby R.K. (2015). The effects of metal ion PCR inhibitors on results obtained with the Quantifiler^®^ Human DNA Quantification Kit. Forensic Sci. Int. Genet..

[15] McCord B., Pionzio A., Thompson R. (2015). Analysis of the Effect of a Variety of PCR Inhibitors on the Amplification of DNA Using Real Time PCR, Melt Curves and STR Analysis.

[16] Snow E.T., Xu L.S., Kinney P.L. (1993). Effects of nickel ions on polymerase activity and fidelity during DNA replication in vitro. Chem. Biol. Interact..

[17] Sidstedt M., Rådström P., Hedman J. (2020). PCR inhibition in qPCR, dPCR and MPS-mechanisms and solutions. Anal. Bioanal. Chem..

[18] Sutlović D., Gojanović M.D., Andelinović S., Gugić D., Primorac D. (2005). Taq polymerase reverses inhibition of quantitative real time polymerase chain reaction by humic acid. Croat Med. J..

[19] Tsai Y.L., Olson B.H. (1992). Detection of low numbers of bacterial cells in soils and sediments by polymerase chain reaction. Appl. Environ. Microbiol..

[20] Sidstedt M., Steffen C.R., Kiesler K.M., Vallone P.M., Rådström P., Hedman J. (2019). The impact of common PCR inhibitors on forensic MPS analysis. Forensic Sci. Int. Genet..

[21] Dierig L., Schwender M., Wiegand P. (2020). Looking for the pinpoint: Optimizing identification, recovery and DNA extraction of micro traces in forensic casework. Forensic Sci. Int. Genet..

[22] Butler J.M. (2023). Recent advances in forensic biology and forensic DNA typing: INTERPOL review 2019–2022. Forensic Sci. Int. Synerg..

[23] Griffin A., Kirkbride K.P., Henry J., Painter B., Linacre A. (2021). DNA on drugs! A preliminary investigation of DNA deposition during the handling of illicit drug capsules. Forensic Sci. Int. Genet..

[24] Wang H., Qi J., Xiao D., Wang Z., Tian K. (2017). A re-evaluation of dilution for eliminating PCR inhibition in soil DNA samples. Soil Biol. Biochem..

[25] Hedman J., Anders N., Birgitta R., Ricky A., Rådström P. (2009). Improved Forensic DNA Analysis Through the use of Alternative DNA Polymerases and Statistical Modeling of DNA Profiles. Biotechniques.

[26] Hedman J., Dufva C., Norén L., Ansell C., Albinsson L., Ansell R. (2011). Applying a PCR inhibitor tolerant DNA polymerase blend in forensic DNA profiling. Forensic Sci. Int. Genet. Suppl. Ser..

[27] Kermekchiev M.B., Kirilova L.I., Vail E.E., Barnes W.M. (2009). Mutants of Taq DNA polymerase resistant to PCR inhibitors allow DNA amplification from whole blood and crude soil samples. Nucleic Acids Res..

[28] McDonald C., Taylor D., Brinkworth R.S.A., Linacre A. (2024). Developing a Machine Learning ‘Smart’ Polymerase Chain Reaction Thermocycler Part 2: Putting the Theoretical Framework into Practice. Genes.

[29] McDonald C., Taylor D., Linacre A. (2023). Smart PCR leading to improved DNA profiles. Aust. J. Forensic Sci..

[30] McDonald C., Taylor D., Masawi G.M., Khan A.K.A., Leibbrandt R., Linacre A., Brinkworth R.S.A., Machine-Learning D.A. (2024). Part 1: Construction of a Theoretical Framework. Genes.

[31] Fujii K., Fukagawa T., Watahiki H., Mita Y., Kitayama T., Mizuno N. (2018). Ratios and distances of pull-up peaks observed in GlobalFiler kit data. Leg. Med..

[32] Taylor D., Bright J.A., McGovern C., Neville S., Grover D. (2017). Allele frequency database for GlobalFiler™ STR loci in Australian and New Zealand populations. Forensic Sci. Int. Genet..

[33] Koller M. (2016). robustlmm: An R Package for Robust Estimation of Linear Mixed-Effects Models. J. Stat. Softw..

[34] Team R.-C. (2020). R: A Language and Environment for Statistical Computing. https://www.R-project.org/.

[35] Alaeddini R. (2012). Forensic implications of PCR inhibition—A review. Forensic Sci. Int. Genet..

[36] Sidstedt M., Jansson L., Nilsson E., Noppa L., Forsman M., Rådström P., Hedman J. (2015). Humic substances cause fluorescence inhibition in real-time polymerase chain reaction. Anal. Biochem..

[37] Hriciková S., Kožárová I., Hudáková N., Reitznerová A., Nagy J., Marcinčák S. (2023). Humic Substances as a Versatile Intermediary. Life.

[38] Schrader C., Schielke A., Ellerbroek L., Johne R. (2012). PCR inhibitors—occurrence, properties and removal. J. Appl. Microbiol..

[39] Lorenz T.C. (2012). Polymerase chain reaction: Basic protocol plus troubleshooting and optimization strategies. J. Vis. Exp..

[40] Steitz T.A. (1998). A mechanism for all polymerases. Nature.

[41] Karunanathie H., Kee P.S., Ng S.F., Kennedy M.A., Chua E.W. (2022). PCR enhancers: Types, mechanisms, and applications in long-range PCR. Biochimie.

