# Supplementary material for: STR-Uggles: Overcoming Humic Acid Inhibition Using Combined STR & qPCR Kit Chemistries
Source: Genes (Basel). 2025 Nov 3;16(11):1326. doi: 10.3390/genes16111326 (PMC12652367; doi:10.3390/genes16111326)
Supplement: Supplementary file 1 [file genes-16-01326-s001.zip › genes-3915787-supplementary.pdf]

SUPPLEMENTARY MATERIALS:

Supplementary Table S1: The breakdown of the profile quality scores for the STR profiles produced from 500 pg of starting material and the standard GlobalFiler setup for the five concentrations of humic acid trialled. For each humic acid concentration n=5

Humic Acid (ng/μL)	Replicate	Peak Heights (RFU)			Penalties			Profile Quality Score
		Mean	Std Dev	COV	Peak Height	COV	Artefacts	
0 (Standard)	A	2922	1450	0.50	-4.34	-1.08	0	-8.81
	B	7761	3829	0.49	-5.05	-1.07	-2	-11.52
	C	4473	1976	0.44	-3.46	-0.96	0	-7.82
	D	5970	2859	0.48	-3.60	-1.04	-2	-10.04
	E	4726	2061	0.44	-3.42	-0.95	0	-7.76
50	A	1318	1238	0.94	-6.34	-2.04	-22	-33.78
	B	6392	3108	0.49	-3.82	-1.06	-8	-16.27
	C	7653	4377	0.57	-4.93	-1.24	-12	-21.57
	D	8436	4463	0.53	-5.96	-1.15	-8	-18.51
	E	1846	1435	0.78	-5.56	-1.69	-10	-20.65
100	A	6228	3488	0.56	-3.73	-1.22	-18	-26.34
	B	3749	3180	0.85	-3.74	-1.84	-10	-18.98
	C	2968	3005	1.01	-4.30	-2.20	-14	-23.89
	D	4186	2396	0.57	-3.54	-1.24	-6	-14.18
	E	3612	2892	0.80	-3.82	-1.74	-6	-14.96
200	A	409	839	2.05	-7.98	-4.46	-32	-47.83
	B	1438	1664	1.16	-6.15	-2.51	0	-12.07
	C	33	109	3.27	-8.76	-7.10	-22	-41.25
	D	1349	784	0.58	-6.29	-1.26	-4	-14.96
	E	1133	1265	1.12	-6.65	-2.42	-6	-18.47
300	A	128	328	2.56	-8.55	-5.55	-18	-35.50
	B	956	1628	1.70	-6.95	-3.70	-10	-24.05
	C	827	1237	1.50	-7.18	-3.25	-12	-25.83
	D	97	152	1.58	-8.62	-3.42	-18	-33.44
	E	7	24	3.46	-8.81	-7.52	-12	-31.73

Supplementary Table S2: The number of observed alleles, percent allele loss and sub-source likelihood ratios for all STR profiles produced from 500 pg of starting material using the standard GlobalFiler setup for each humic acid concentration trialled. For all profiles the expected number of donor alleles was 37. Replicates that did not meet the requirements for upload to the National Criminal Identification Database (NCIDD) have been marked with an asterisk (*).

Humic Acid (ng/μL)	Replicate	Number of Observed Alleles	Average Percent Allele Loss (%)	Sub-Source Likelihood Ratio
0 (Standard)	A	37	0.00	2.26×10^{26}
	B	37	0.00	2.26×10^{26}
	C	37	0.00	2.26×10^{26}
	D	37	0.00	2.26×10^{26}
	E	37	0.00	2.26×10^{26}
50	A	37	0.00	2.12×10^{26}
	B	37	0.00	2.26×10^{26}
	C	37	0.00	2.26×10^{26}
	D	37	0.00	2.26×10^{26}
	E	37	0.00	2.26×10^{26}
100	A	37	0.00	5.62×10^{25}
	B	37	0.00	1.55×10^{25}
	C	37	0.00	5.62×10^{25}
	D	37	0.00	2.26×10^{26}
	E	37	0.00	2.26×10^{26}
200	A	14	62.16	1.45×10^{5}
	B	37	0.00	2.26×10^{26}
	C	6*	83.78	8.16×10^{2}
	D	37	0.00	2.26×10^{26}
	E	37	0.00	2.25×10^{26}
300	A	8*	78.38	1.30×10^{5}
	B	28	24.32	1.45×10^{19}
	C	22	40.54	5.01×10^{13}
	D	17	54.05	3.57×10^{6}
	E	3*	91.89	1.32×10^{1}

Supplementary Table S3: The breakdown of the profile quality scores for the STR profiles produced from 500 pg of starting material and the combined GlobalFiler and Investigator Quantiplex Pro setup for the five concentrations of humic acid trialled. For each humic acid concentration n=5

Humic Acid (ng/μL)	Replicate	Peak Heights (RFU)			Penalties			Profile Quality Score
		Mean	Std Dev	COV	Peak Height	COV	Artefacts	
0 (Standard)	A	7000	4615	0.66	-4.27	-1.43	0	-9.10
	B	7831	4351	0.56	-5.14	-1.21	-4	-13.75
	C	7507	4067	0.54	-4.76	-1.18	-2	-11.34
	D	8534	4680	0.55	-6.11	-1.19	-4	-14.70
	E	6317	3547	0.56	-3.78	-1.22	0	-8.39
50	A	4786	4660	0.97	-3.41	-2.11	-8	-16.92
	B	7694	4405	0.57	-4.97	-1.24	-2	-11.62
	C	8180	4433	0.54	-5.60	-1.18	0	-10.17
	D	7532	4686	0.62	-4.79	-1.35	-6	-15.54
	E	7051	4080	0.58	-4.31	-1.26	-2	-10.97
100	A	7537	4752	0.63	-4.80	-1.37	-6	-15.56
	B	4726	4138	0.88	-3.42	-1.90	-16	-24.72
	C	4051	3344	0.83	-3.59	-1.79	-12	-20.79
	D	4647	3676	0.79	-3.43	-1.72	-10	-18.54
	E	6795	3888	0.57	-4.10	-1.24	-8	-16.74
200	A	8224	5299	0.64	-5.66	-1.40	-10	-20.45
	B	4629	3338	0.72	-3.43	-1.57	-8	-16.39
	C	7460	4160	0.56	-4.71	-1.21	-4	-13.32
	D	4866	3499	0.72	-3.40	-1.56	-10	-18.36
	E	6626	4055	0.61	-3.97	-1.33	-14	-22.70
300	A	3707	3824	1.03	-3.76	-2.24	-20	-29.40
	B	5595	4105	0.73	-3.48	-1.59	-12	-20.47
	C	101	258	2.55	-8.61	-5.53	-22	-39.54
	D	5722	3913	0.68	-3.51	-1.49	-2	-10.40
	E	285	557	1.95	-8.23	-4.24	-24	-39.86

Supplementary Table S4: The number of observed alleles, percent allele loss and sub-source likelihood ratios for all STR profiles produced from 500 pg of starting material using the combined GlobalFiler and Investigator Quantiplex Pro setup for each humic acid concentration trialled. For all profiles the expected number of donor alleles was 37. Replicates that did not meet the requirements for upload to the National Criminal Identification Database (NCIDD) have been marked with an asterisk (*).

**Humic Acid (ng/μL)**	**Replicate**	**Number of Observed Alleles**	**Average Percent Allele Loss (%)**	**Sub-Source Likelihood Ratio**
0 (Standard)	A	37	0.00	2.26×10^{26}
	B	37	0.00	2.26×10^{26}
	C	37	0.00	2.26×10^{26}
	D	37	0.00	5.62×10^{25}
	E	37	0.00	2.26×10^{26}
50	A	37	0.00	5.62×10^{25}
	B	37	0.00	2.36×10^{24}
	C	37	0.00	2.26×10^{26}
	D	37	0.00	5.62×10^{25}
	E	37	0.00	5.62×10^{25}
100	A	37	0.00	2.26×10^{26}
	B	37	0.00	5.62×10^{25}
	C	37	0.00	2.26×10^{26}
	D	37	0.00	2.26×10^{26}
	E	37	0.00	2.26×10^{26}
200	A	37	0.00	2.26×10^{26}
	B	37	0.00	2.26×10^{26}
	C	37	0.00	2.26×10^{26}
	D	37	0.00	5.62×10^{25}
	E	37	0.00	2.26×10^{26}
300	A	34	8.11	9.64×10^{21}
	B	37	2.70	6.66×10^{24}
	C	8*	75.68	1.30×10^{5}
	D	37	0.00	2.26×10^{26}
	E	15	59.46	1.77×10^{8}

Supplementary Table S5: The breakdown of the profile quality scores for the STR profiles produced from 500 pg of starting material with the GlobalFiler with additional polymerase and buffer setup for the five concentrations of humic acid trialled. For each humic acid concentration n=5, except 300 ng of humic acid where n=4 (one replicate failed to produce any profile).

Humic Acid (ng/μL)	Replicate	Peak Heights (RFU)			Penalties			Profile Quality Score
		Mean	Std Dev	COV	Peak Height	COV	Artefacts	
0 (Standard)	A	7415	4670	0.63	-4.67	-1.37	-2	-11.43
	B	4981	3296	0.66	-3.40	-1.44	0	-8.24
	C	6303	3687	0.59	-3.77	-1.27	0	-8.44
	D	7387	4597	0.62	-4.64	-1.35	-6	-15.39
	E	6250	3876	0.62	-3.74	-1.35	0	-8.48
50	A	7278	4272	0.59	-4.53	-1.27	0	-9.20
	B	6012	4430	0.74	-3.62	-1.60	0	-8.62
	C	842	1654	1.96	-7.15	-4.27	-2	-16.82
	D	2007	2016	1.00	-5.34	-2.18	0	-10.93
	E	6537	4439	0.68	-3.91	-1.47	0	-8.79
100	A	4047	3803	0.94	-3.60	-2.04	-14	-23.04
	B	4575	3101	0.68	-3.44	-1.47	0	-8.31
	C	5216	4319	0.83	-3.41	-1.80	-2	-10.61
	D	5366	4346	0.81	-3.43	-1.76	-2	-10.59
	E	4837	4240	0.88	-3.40	-1.90	-4	-12.71
200	A	2519	3636	1.44	-4.74	-3.13	-4	-15.27
	B	3053	3023	0.99	-4.22	-2.15	-6	-15.77
	C	5058	3925	0.78	-3.40	-1.68	-4	-12.48
	D	6011	3876	0.64	-3.62	-1.40	-2	-10.42
	E	6938	4523	0.65	-4.21	-1.42	0	-9.03
300	A	4116	4308	1.05	-3.57	-2.27	-2	-11.24
	B	2888	3877	1.34	-4.37	-2.92	-4	-14.68
	C	1514	2809	1.86	-6.04	-4.03	-2	-15.47
	E	2671	3026	1.13	-4.58	-2.46	0	-10.44

Supplementary Table S6: The number of observed alleles, percent allele loss and sub-source likelihood ratios for all STR profiles produced from 500 pg of starting material with the GlobalFiler with additional polymerase and buffer setup for each humic acid concentration trialled. For all profiles the expected number of donor alleles was 37. Replicates that did not meet the requirements for upload to the National Criminal Identification Database (NCIDD) have been marked with an asterisk (*).

Humic Acid (ng/μL)	Replicate	Number of Observed Alleles	Average Percent Allele Loss (%)	Sub-Source Likelihood Ratio
0 (Standard)	A	37	0	2.26×10^{26}
	B	37	0	2.26×10^{26}
	C	37	0	2.26×10^{26}
	D	37	0	2.26×10^{26}
	E	37	0	2.26×10^{26}
50	A	37	0	2.26×10^{26}
	B	37	0	2.26×10^{26}
	C	15	59.50	2.24×10^{9}
	D	32	13.51	2.07×10^{22}
	E	37	0	2.26×10^{26}
100	A	35	5.41	4.10×10^{23}
	B	37	0	2.26×10^{26}
	C	37	0	2.26×10^{26}
	D	36	2.70	1.06×10^{25}
	E	35	5.41	4.10×10^{23}
200	A	22	40.54	5.19×10^{13}
	B	32	13.51	6.37×10^{21}
	C	37	0	2.26×10^{26}
	D	37	0	2.26×10^{26}
	E	37	0	2.26×10^{26}
300	A	30	18.92	7.20×10^{20}
	B	24	35.14	9.70×10^{11}
	C	15	59.46	6.83×10^{9}
	E	30	18.92	7.20×10^{20}

Supplementary Table S7: The breakdown of the profile quality scores for the STR profiles produced from 500 pg of starting material and the GlobalFiler with additional qPCR polymerase and buffer setup for the five concentrations of humic acid trialled. For each humic acid concentration n=5

Humic Acid (ng/μL)	Replicate	Peak Heights (RFU)			Penalties			Profile Quality Score
		Mean	Std Dev	COV	Peak Height	COV	Artefacts	
0 (Standard)	A	10145	5646	0.56	-9.15	-1.21	0	-13.75
	B	7082	4204	0.59	-4.34	-1.29	0	-9.03
	C	2840	2685	0.95	-4.41	-2.05	0	-9.86
	D	6461	3969	0.61	-3.86	-1.33	0	-8.60
	E	8585	4653	0.54	-6.19	-1.18	0	-10.77
50	A	9051	4825	0.53	-6.96	-1.16	0	-11.52
	B	8247	4509	0.55	-5.69	-1.19	0	-10.27
	C	7704	4522	0.59	-4.99	-1.27	0	-9.66
	D	9153	5064	0.55	-7.14	-1.20	0	-11.74
	E	8125	4456	0.55	-5.52	-1.19	0	-10.11
100	A	8029	4327	0.54	-5.39	-1.17	0	-9.96
	B	4438	2896	0.65	-3.47	-1.42	0	-8.28
	C	9261	4699	0.51	-7.34	-1.10	-2	-13.84
	D	7722	4324	0.56	-5.01	-1.22	-2	-11.62
	E	9187	5015	0.55	-7.21	-1.19	0	-11.79
200	A	8559	5003	0.58	-6.15	-1.27	0	-10.82
	B	6928	4118	0.59	-4.21	-1.29	0	-8.90
	C	6329	3860	0.61	-3.78	-1.32	-2	-10.51
	D	8464	4780	0.56	-6.00	-1.23	0	-10.63
	E	7842	4273	0.54	-5.15	-1.18	-2	-11.74
300	A	7942	4077	0.51	-5.28	-1.11	0	-9.79
	B	3814	4246	1.11	-3.70	-2.42	0	-9.52
	C	4754	4642	0.98	-3.41	-2.12	0	-8.93
	D	6232	4613	0.74	-3.73	-1.61	-4	-12.74
	E	4947	4492	0.91	-3.40	-1.97	0	-8.77

Supplementary Table S8: The number of observed alleles, percent allele loss and sub-source likelihood ratios for all STR profiles produced from 500 pg of starting material using the GlobalFiler with additional qPCR polymerase and buffer setup for each humic acid concentration trialled. For all profiles the expected number of donor alleles was 37. Replicates that did not meet the requirements for upload to the National Criminal Identification Database (NCIDD) have been marked with an asterisk (*).

**Humic Acid (ng/μL)**	**Replicate**	**Number of Observed Alleles**	**Average Percent Allele Loss (%)**	**Sub-Source Likelihood Ratio**
0 (Standard)	A	37	0	2.26×10^{26}
	B	37	0	2.26×10^{26}
	C	37	0	2.26×10^{26}
	D	37	0	2.26×10^{26}
	E	37	0	2.26×10^{26}
50	A	37	0	2.26×10^{26}
	B	37	0	2.26×10^{26}
	C	37	0	2.26×10^{26}
	D	37	0	2.26×10^{26}
	E	37	0	2.26×10^{26}
100	A	37	0	2.26×10^{26}
	B	37	0	2.26×10^{26}
	C	37	0	2.26×10^{26}
	D	37	0	2.26×10^{26}
	E	37	0	2.26×10^{26}
200	A	37	0	2.26×10^{26}
	B	37	0	2.26×10^{26}
	C	37	0	2.26×10^{26}
	D	37	0	2.26×10^{26}
	E	37	0	2.26×10^{26}
300	A	37	0	2.26×10^{26}
	B	30	18.92	7.21×10^{20}
	C	35	5.41	3.70×10^{23}
	D	37	0	2.26×10^{26}
	E	36	2.70	1.04×10^{25}
